# Speed and Duration of Walking and Other Leisure Time Physical Activity and the Risk of Heart Failure: A Prospective Cohort Study from the Copenhagen City Heart Study

**DOI:** 10.1371/journal.pone.0089909

**Published:** 2014-03-12

**Authors:** Hans Askelund Saevereid, Peter Schnohr, Eva Prescott

**Affiliations:** 1 Department of Cardiology, Bispebjerg University Hospital, Copenhagen, Denmark; 2 The Copenhagen City Heart Study, Frederiksberg Hospital, Copenhagen, Denmark; University College London, United Kingdom

## Abstract

**Aim:**

Physical activity (PA) confers some protection against development of heart failure (HF) but little is known of the role of intensity and duration of exercise.

**Methods and Results:**

In a prospective cohort study of men and women free of previous MI, stroke or HF with one or more examinations in 1976–2003, we studied the association between updated self-assessed leisure-time PA, speed and duration of walking and subsequent hospitalization or death from HF. Light and moderate/high level of leisure-time PA and brisk walking were associated with reduced risk of HF in both genders whereas no consistent association with duration of walking was seen. In 18,209 subjects age 20–80 with 1580 cases of HF, using the lowest activity level as reference, the confounder-adjusted hazard ratios (HR) for light and moderate/high leisure-time physical activity were 0.75 (0.66–0.86) and 0.80 (0.69–0.93), respectively. In 9,937 subjects with information on walking available and 542 cases of HF, moderate and high walking speed were associated with adjusted HRs of 0.53 (0.43–0.66) and 0.30 (0.21–0.44), respectively, and daily walking of ½–1 hrs, 1–2 and >2 hrs with HR of 0.80 (0.61–1.06), 0.82 (0.62–1.06), and 0.96 (0.73–1.27), respectively. Results were similar for both genders and remained robust after exclusion of HF related to coronary heart disease and after a series of sensitivity analyses.

**Conclusions:**

Speed rather than duration of walking was associated with reduced risk of HF. Walking is the most wide-spread PA and public health measures to curb the increase in HF may benefit from this information.

## Introduction

With aging of the population chronic heart failure (HF) has become a major health issue throughout the world. The prevalence of heart failure is approximately 1–2% in the western world reaching ≥10% among persons 70 years of age or older [1]. Despite better treatment, the high costs, morbidity and mortality due to HF remains an issue, and the prevention of HF deserves high priority. Improved knowledge on the effects of physical activity (PA), including intensity and duration, on development of HF is important for clarifying potential mechanisms and for providing guidance on PA recommendations in prevention of HF.

Walking is the most common, popular and accessible mode of PA [2]–[5] and walking has been shown to have beneficial effects on cardiovascular disease (CVD) [6], [7]. Recent evidence suggests that speed rather than duration of the PA has a stronger effect on the risk of CVD [6]–[8], coronary heart disease (CHD) [9], [10], and all-cause mortality [7], [11]. Several studies have also found that PA is associated with reduced risk of developing HF [12]–[18], but there are no studies of the effects of walking or that differentiate between the effects of intensity and duration.

Our study aims to assess the association between walking and other leisure time PA and HF in a large population study with repeated examination and more than 30 years follow-up with emphasis on the independent effects of speed and duration of walking.

## Methods

### Study population

**Figure 1:**
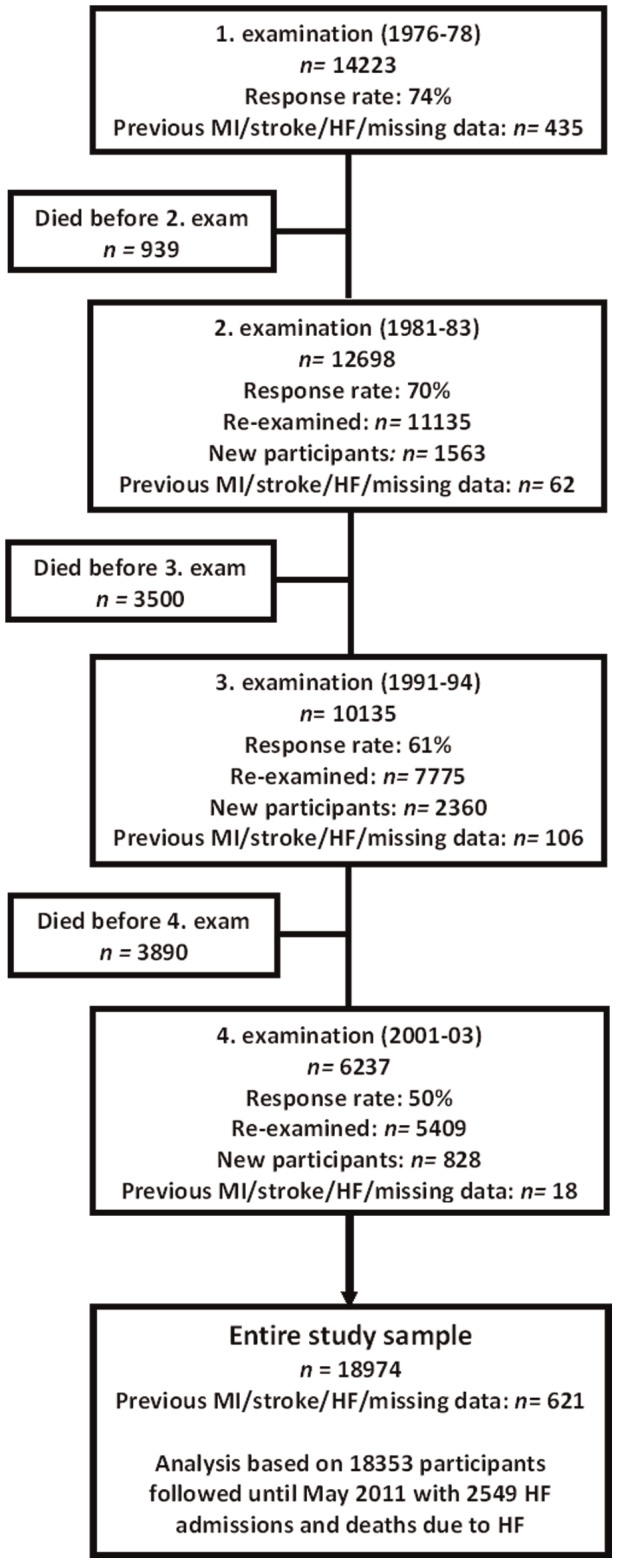
Flow diagram of the study population. The entire study sample consisted of persons participating in at least one of the four examinations in the Copenhagen City Heart Study who were free of previous myocardial infarction, heart failure and stroke at their first examination in the study. n, numbers.

In the Copenhagen City Heart Study (CCHS), an ongoing population study, a random sample of the population living in a specific area of Copenhagen, Denmark, have since 1976–78 been invited to participate at regular intervals. Details have been described elsewhere [19]. The original sample included 14,223 participants. In 1981–83, 1991–94 and 2001–3, participants were re-examined and new, primarily younger subjects were invited. A total of 18,974 subjects have participated in one or more of the examinations (*Figure 1*). After excluding 621 participants with missing values on leisure-time PA (LTPA) or with previous MI, HF or stroke according to the Danish National Patient Register prior to their study inclusion, this study included a total of 18,353 participants. Cardiovascular risk factors were assessed at each of the four examinations using a self-administered questionnaire, a physical examination, and blood samples. Information on LTPA was available at all four examinations (n = 18,353) whereas information on speed and duration of walking was available from the third examination onward, which limited the number of participants to 10,411. Danish legislation does not require ethical approval for registry linkage. A written informed consent was obtained from the participants in the two most recent examinations. Further, a verbal informed consent was obtained from the two first examinations, as the written consent was not invented at the time. The Ethics Committee for the Copenhagen area approved the study (KF 100.2039/91).

### Leisure-time physical activity variables

LTPA, from The CCHS Leisure Time PA Questionnaire [20], was self-assessed according to four pre-specified categories (1) *Sedentary*: sedentary or light PA (e.g. slow walking/biking) <2 hours/week, (2) *Light PA*: light PA 2–4 h/week, (3) *Moderate PA*: light PA >4 h/week or 2–4 h/week of more vigorous PA (e.g. brisk walking, fast biking) and (4) *High PA*: more than 4 h/week of moderate PA or regular heavy exercise or competitive sports several times per week. Walking duration and speed were self-reported as *never, <½ hour, ½–1 hour, 1–2 hours and >2 hours per day* and *low, moderate, high or very high speed*, respectively. To avoid inadequate group sizes the *high* and *very high* walking speed categories, and *never* and *<½ hour* duration categories were merged. In the same way LTPA groups 3 and 4 were merged.

### Covariates

Covariates under consideration were classified as potential confounders, indicators of co-morbidity and mediators. Age, gender, smoking (never smoker, ex-smoker, 1–15 cigarettes per day, 15–24 cigarettes per day, or >24 cigarettes per day), alcohol consumption (abstainers, monthly, weekly, or daily intake), education (<8, 8–10 and >10 years, corresponding to lower primary school, higher primary school and secondary school, respectively), household income (low, medium, and high) and family history of CVD were self-reported and considered confounders in the association between PA and HF. Atrial fibrillation, impaired lung function and diabetes (self-reported) were considered indicators of co-morbidity. Lung function was measured as the observed forced expiratory volume (FEV_1_) in % of the predicted FEV_1_ based on gender, age and height [21]. Presence of atrial fibrillation was determined through ECG findings and was coded according to the Minnesota Code Classification System for Electrocardiographic. Systolic blood pressure, heart rate, total cholesterol, triglycerides, and body mass index (BMI) were considered partial mediators, i.e. part of the causal pathway between PA and HF. Blood samples were drawn non-fasting. Total cholesterol was available at all examinations and triglycerides at all but the second. Heart rate, partially perceived as an indicator of physical fitness, was measured with ECG and systolic blood pressure was measured (by a London School of Hygiene sphygmomanometer) in a sitting position after 5 minutes rest. BMI was calculated as weight (kg) divided by height squared (m^2^). Treatment for hypertension was self-reported.

### Endpoints

The primary endpoints were first hospital admission with a diagnosis of HF (ICD8 codes 425.99, 427.09–427.11, 427.19, and 428.99 until 1st January 1994 and ICD10 codes I11.0, I25.5, I42.0, I42.6, I42.9, I50.0-9 from 1994 and onwards) ascertained from the Danish National Patient Registry, which has registered all admissions to all Danish hospitals since 1977, and death due to HF from the Danish Register of Causes of Death. Follow-up was until first admission or death from HF, death from other causes, emigration (<0.5%) or end of follow-up (May 2011), whichever came first.

### Analytical strategy

The primary exposure variables were level of PA during leisure time and speed and duration of walking. Comparison across the different groups defined by level of PA was performed by means of Chi2-test for the categorical data and one-way analysis of variance (ANOVA) for the continuous data. Because of age-differences, they were also compared by linear and logistic regression adjusting for age. A two-sided p-value of 0.05 was considered significant.

Hazard ratios (HRs) were used to describe the effect of level of LTPA and speed- and duration of walking on the risk of HF, and were estimated with Cox proportional hazards regression models, with age as the underlying timescale and delayed entry to ensure an optimal adjustment for age. In all the main analyses, updated information on LTPA, speed- and duration of walking, confounders, comorbidity parameters and mediating factors were used whenever these were available (i.e. time-dependent variables). Test for interaction was by log-likelihood test comparing a model with the interaction term with a model without.

The analytical strategy was initially to adjust for age, then for confounders including co-morbidity parameters and finally a multivariable analysis including confounders, co-morbidity parameters and potential mediators. A global Chi-squared test based on Schoenfeld residuals to verify the assumption of proportional hazards was conducted. The assumption was violated with regard to several of the exposure variables and survival analyses were therefore restricted to age below 80 years at study entry and censoring of follow-up time when the participant reached age 80. This reduced number of participants from 18,353 to 18,209, number of outcomes from 2,549 to 1,580 and person-years under observation from 385,129 to 353,502 in CCHS part 1–4 (analyses of LTPA) and number of participants from 10,411 to 9,937, number of outcomes from 1063 to 541 and person-years under observation from 140,890 to 122,981 in CCHS part 3–4 (analyses of walking speed and duration). With this restriction, model assumptions were not violated.

A series of sensitivity analyses were performed: To avoid any influence of subclinical disease (reverse causation) the first two years of follow-up were excluded. The role of co-morbidity was assessed by excluding participants with a self-reported walking disability (n = 1741) from analyses on walking speed and duration. Approximately 11% of cases of the outcomes were death from HF not preceded by hospital admission. Because the cause of death in this case is likely to be less valid, analyses were repeated treating these cases as censored. Analyses were also repeated using only baseline data (i.e. no time-dependent variables) and, further, allowing for the competing risk from mortality from any cause based on the method of Fine and Gray [22] and after excluding participants reporting antihypertensive medication. Finally, information on proBNP was available only from the fourth examination and analysis adjusting for proBNP was therefore repeated based only on this study.

More than 50% of HF is caused by CHD [1]. To determine whether any associations with PA differ between HF caused by CHD and from other aetiology, analyses were repeated after excluding all cases of HF in subjects who at any time during follow-up had a hospital admission for acute coronary syndrome (ICD8: 410; ICD10: I21-I22).

Data was analysed using Stata version 11.2 (Stat Corp. LP, College Station, TX, USA).

## Results

### Baseline data

The baseline characteristics for the 8,422 men and 9,931 women are displayed in *Table 1*. In general, higher level of PA was associated with better cardiovascular risk factor. Notably, after age-adjustment, systolic blood pressure did not differ significantly between groups. Similar associations with CVD risk factors were found for walking speed and duration. There was a clear trend in declining heart rate, an indicator of physical fitness, from the sedentary to the moderate/highly active participants and with increasing speed of walking in both genders (all age-adjusted test for trend <0.001) whereas heart rate did not differ between groups defined by walking duration (age-adjusted test for trend >0.10). (results not shown).

**Table 1:** Risk factor profile by level of physical activity among 8422 men and 9931 women in the Copenhagen City Heart Study.

	Men		Women	
	Sedentary	Light	Moderate/High	p-value^a^	Sedentary	Light	Moderate/High	p-value^a^
	n = 1528	n = 3875	n = 3019		n = 1799	n = 5574	n = 2558	
*Confounders*								
Age (years)	53.6 (12.9)	51.4 (12.8)	46.9 (14.3)		54.3 (12.5)	50.5 (12.8)	46.3 (14.4)	
Current smoker	1131 (74.0)	2672 (69.0)	1778 (59.0)	<0.001	1139 (63.3)	3087 (55.4)	1339 (52.4)	<0.001
Daily alcohol consumption	625 (41.1)	1383 (35.9)	883 (29.5)	<0.001	154 (8.6)	276 (5.0)	105 (4.1)	<0.001
Family history of CVD	432 (32.1)	1127 (31.3)	754 (26.5)	0.006	588 (35.8)	1896 (35.9)	759 (30.8)	0.41
Low education	816 (53.6)	1585 (40.9)	1001 (33.2)	<0.001	1033 (57.7)	2244 (40.3)	780 (30.5)	<0.001
Low household income	488 (32.2)	840 (21.9)	653 (21.8)	<0.001	753 (45.0)	1608 (30.2)	739 (30.1)	<0.001
*Co-morbidity parameters*								
Atrial fibrillation	13 (0.9)	30 (0.8)	8 (0.3)	0.12	19 (1.1)	16 (0.3)	8 (0.3)	0.12
Diabetes	87 (6.1)	129 (3.5)	77 (2.7)	<0.001	47 (2.7)	103 (1.9)	24 (1.0)	0.003
Lung function % pred	76.4 (20.4)	81.8 (19.2)	86.8 (17.3)	<0.001	80.8 (20.5)	86.7 (17.7)	89.7 (16.8)	<0.001
*Mediators*								
Triglycerides (mmol/L)	2.2 (1.5)	2.1 (1.4)	1.9 (1.3)	<0.001	1.6 (1.0)	1.5 (0.8)	1.3 (0.9)	<0.001
Total cholesterol (mmol/L)	5.9 (1.2)	5.9 (1.2)	5.6 (1.2)	<0.001	6.3 (1.3)	6.1 (1.3)	5.8 (1.3)	0.003
Systolic blood-pressure (mmHg)	140.3 (21.0)	139.4 (20.8)	136.0 (19.2)	0.84	136.5 (23.2)	132.5 (22.3)	128.6 (21.1)	0.32
Treated hypertension	92 (6.1)	211 (5.5)	93 (3.1)	0.009	173 (9.7)	339 (6.1)	120 (4.7)	<0.001
Heart rate (b.p.m.)	75.3 (13.5)	74.2 (13.1)	70.8 (12.8)	<0.001	75.9 (12.9)	73.7 (12.1)	71.9 (12.1)	<0.001
BMI (kg/m^2)^	26.2 (4.1)	25.7 (3.6)	25.6 (3.5)	0.002	25.1 (4.9)	24.6 (4.3)	24.1 (3.8)	<0.001

Categorical data displayed as numbers (%) and continuous data displayed as means (SD).

a *P*-values age-adjusted by means of logistic regression for categorical data and linear regression for continuous data.

Lung function % pred: FEV_1_ in % of the predicted FEV_1_ based on gender, age and height.

### Leisure-time physical activity and risk of HF

Age-adjusted HRs for men with a light or moderate/high level of PA were 0.61 (0.51–0.73) and 0.64 (0.54–0.77), respectively, compared to a sedentary level (*Table 2*). Results for women were similar with corresponding HRs of 0.59 (0.50–0.71) and 0.50 (0.41–0.63). HRs were attenuated by adjustment for confounding factors and further after adjustment for mediating factors but remained of borderline statistical significance, albeit with no clear dose response relationship.

**Table 2:** Hospital admission or death from heart failure by duration and speed of walking and other leisure time physical activity.

		Men		Women
	No. of endpoints	HR^a^	HR^b^	HR^c^	No. of endpoints	HR^s^	HR^b^	HR^c^
***Leisure- time physical activity*** d								
Sedentary	182	1 (ref.)	1 (ref.)	1 (ref.)	182	1 (ref.)	1 (ref.)	1 (ref.)
Light	354	0.61 (0.51–0.73)	0.76 (0.63–0.91)	0.78 (0.64–0.94)	365	0.59 (0.50–0.71)	0.77 (0.64–0.92)	0.85 (0.70–1.03)
Moderate/High	310	0.64 (0.54–0.77)	0.87 (0.72–1.06)	0.91 (0.75–1.10)	149	0.50 (0.41–0.63)	0.72 (0.57–0.90)	0.81 (0.64–1.04)
*p-value for linear trend*		*<0.001*	*0.48*	*0.75*		*<0.001*	*0.004*	*0.09*
***Duration of daily walking*** e								
Never-½hour	48	1 (ref.)	1 (ref.)	1 (ref.)	41	1 (ref.)	1 (ref.)	1 (ref.)
½hour–1 hour	82	0.82 (0.57–1.17)	0.90 (0.62–1.29)	0.95 (0.66–1.37)	72	0.61 (0.41–0.89)	0.68 (0.46–1.03)	0.73 (0.49–1.10)
1 hour–2 hours	63	0.68 (0.47–0.99)	0.76 (0.52–1.12)	0.81 (0.55–1.20)	80	0.67 (0.46–0.97)	0.76 (0.52–1.12)	0.93 (0.62–1.39)
>2 hours	80	1.04 (0.72–1.48)	1.1 (0.77–1.60)	1.18 (0.82–1.71)	50	0.63 (0.42–0.96)	0.78 (0.51–1.20)	0.84 (0.55–1.30)
*p-value for linear trend*		*0.80*	*0.61*	*0.41*		*0.15*	*0.74*	*0.98*
***Speed of walking*** e								
Low	59	1 (ref.)	1 (ref.)	1 (ref.)	65	1 (ref.)	1 (ref.)	1 (ref.)
Moderate	194	0.39 (0.29–0.53)	0.49 (0.36–0.66)	0.52 (0.38–0.71)	158	0.42 (0.32–0.57)	0.59 (0.43–0.80)	0.64 (0.47–0.88)
High	20	0.18 (0.11–0.30)	0.28 (0.16–0.47)	0.33 (0.19–0.57)	20	0.20 (0.12–0.32)	0.34 (0.20–0.58)	0.41 (0.24–0.71)
*p-value for linear trend*		*<0.001*	*<0.001*	*<0.001*		*<0.001*	*<0.001*	*<0.001*

a Adjusted for age.

b Adjusted for confounders and co-morbidity parameters as described in methods.

c Adjusted for confounders and potential mediators as described in methods.

d Based on 18,209 participants from CCHS part 1–4 with information on LTPA.

e Based on the 9,937 participants from CCHS part 3 and 4 with information on walking speed and duration.

### Duration of walking

Duration of walking was associated with the risk of HF in women, but not in men *(* *Table 2* *)*, *w*ith no indication of a dose dependency, and after adjustment for confounders, no associations were seen.

### Speed of walking

**Figure 2:**
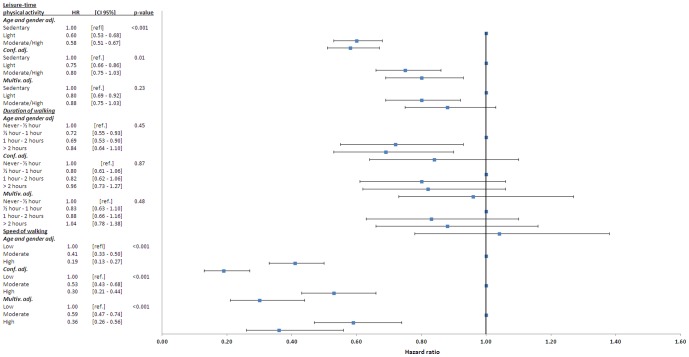
Hazard ratios for HF according to self-assessed duration- and speed of walking. From analyses pooling men and women free of previous heart failure, myocardial infarction and stroke at baseline. Conf. adj. – adjusted for confounders as specified in methods. Multiv. adj. – adjusted for confounders and potential mediators as described in methods. *p-value* for linear trend.

There was a clear dose-response associations between speed of walking and the risk of HF reaching a HR of 0.18 (0.11–0.30) and 0.20 (0.12–0.32) for the group characterized by high walking speed in men and women, respectively. The association between brisk walking and the subsequent risk of developing HF was attenuated but remained highly significant after adjustment for confounders and potential mediators in both genders *(* *Table 2* *)*. Since all associations were similar in men and women, the analyses was repeated on gender-pooled data as shown in *Figure 2*. Only speed of walking was strongly associated with HF in a dose-dependent manner reaching a HR of 0.36 (0.26–0.56) for high speed of walking after multivariable adjustment.

**Figure 3:**
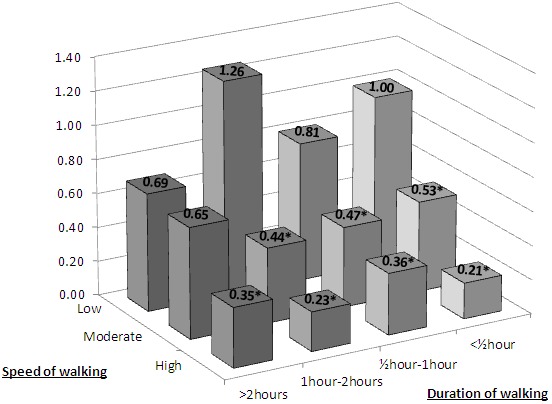
Hazard ratios for HF by categories defined by self-assessed speed and duration of walking. Adjusted for confounders as described in methods. * p<0.05.

After further adjustment for duration of walking, the multivariable adjusted HR for moderate and high speed was 0.57 (0.46–0.72) and 0.37 (0.25–0.55), respectively (p for trend <0.001), with no interaction between the effects of speed and duration (p for interaction 0.30). The adjusted HR in categories defined by speed and duration of walking are shown in *Figure 3*. These confirm an inverse dose-response relationship between speed of walking within each stratum defined by walking duration but no protective effect of duration of walking on risk of subsequent HF within each stratum defined by walking speed.

**Figure 4:**
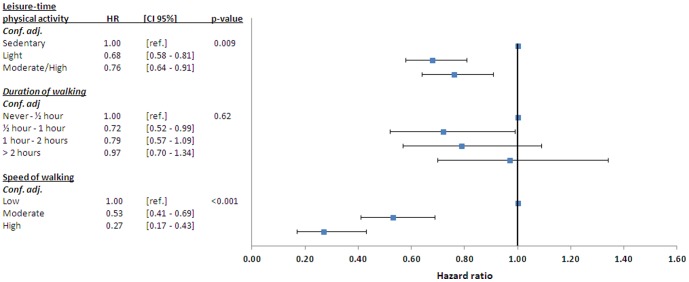
Hazard ratios of heart failure according to self-assessed duration- and speed of walking. From analyses pooling men and women after exclusion of participants admitted to hospital with diagnosis of acute coronary syndrome during follow-up. Conf. adj. - Adjusted for confounders as described in methods. *p-value* for linear trend.

To determine whether effect of PA is through the well-established linkage between exercise and ischemic heart disease, analyses were repeated after excluding subjects admitted for acute coronary syndrome at any time during follow-up as described. This reduced the number of outcomes by approximately 30%, but associations between remained similar (*Figure 4*).

A series of sensitivity analyses were performed as described in the methods section. These all yielded similar results [Tables S1–S6].

## Discussion

The main finding in this large prospective population study with more than 30 years follow-up is an inverse dose-response relationship between PA and risk of HF. Although causality may not be inferred, the results suggest that intensity rather than duration may be important for risk reduction with the lowest risks seen in those reporting high speeds of walking. The results were not explained by confounding by a number of known risk factors and remained robust after a series of sensitivity analyses.

### Comparison with the published literature

Current knowledge about the association of PA and HF is limited and none of the prospective studies reporting on the association between PA and HF have differentiated between duration and intensity of the exercise. In a large Finnish cohort [16], [18] (n∼58,000, 3508 cases of HF) the multivariate adjusted HR associated with high versus low leisure-time PA were 0.66 (0.55–0.79) and 0.74 (0.59–0.92) in men and women, respectively. In the Physician's Health Study (∼21000 men, 1200 cases of HF) lifetime risk of HF for a 40 year old male engaging in exercise > = 5 times weekly was 11.4% versus 14.3% in those exercising less [12]. Self-reported vigorous activity defined as enough to work up a sweat 5 to 7 times a week was associated with a HR of 0.73 (0.59–0.90) comparing with rarely/never PA [17]. In the NHANES (n∼13600, 1382 HF cases) a lower level of recreational PA was associated with a HR for hospital admission or death from HF after multivariable adjustment of 0.88 (0.72–1.06) in men and 0.76 (0.65–0.90) in women [15]. Two recent studies of elderly subjects have reported a dose response relationship between PA and subsequent risk of HF: In the Framingham Heart Study (n∼1142, mean age 76, 250 HF cases,) the highest tertile of PA was associated with an adjusted HR of 0.65 (0.46–0.91) with similar results for HF with preserved and reduced ejection fraction [13], and in the Cardiovascular Health Study (n∼5503, mean age 73) the highest of four PA categories was associated with a HR of 0.60 [14].

In contrast to HF, there are several studies comparing the effects of intensity and duration of exercise on CHD, including studies of duration and speed of walking. In the Health Professionals Follow-up Study (44,000 men followed from 1986 to 1998) half an hour of walking daily was associated with 18% risk reduction and walking pace was associated with reduced risk independently of walking volume. Additionally, time spent walking was not materially associated with CHD risk in analyses that controlled for walking pace [9]. This was supported recently by data from the MORGEN study, following more than 17,000 participants for 9.8 years, which showed no association between duration of walking and the risk of CVD, although it was suggested that the most likely explanation for not finding an association was that almost everybody walked [8]. From a recent meta-analysis including 18 prospective studies with a total of 450.000 participants and 19.000 cases of CVD, a subgroup analysis concluded that both longer duration and higher speed of walking are associated with a reduced risk of CVD in both genders , but that walking speed is a stronger independent predictor of overall risk compared with walking volume with risk reductions of 48% and 26%, respectively [7].

The current study is, to our knowledge, the first large prospective study to compare volume and intensity of PA on the risk of both ischaemic and non-ischaemic HF and our results are in line with those on CVD. The underlying mechanisms which provide a preventive effect on HF through PA are likely to be multiple. Exercise has in small studies been shown to partially prevent age-related cardiac remodelling and is associated with better cardiac function function [23]–[25]. PA has a beneficial effect on obesity [26], diabetes [27], [28], lipid profile [29], and reduces the risk of hypertension [30], all important clinical risk factors in the development of both ischemic and non-ischemic HF [31]. We recently found in a follow-up on the Copenhagen City Heart Study that both leisure-time PA, speed of walking and jogging were protective against a clustering of these risk factors in the metabolic syndrome (MS) while no effect was seen for duration of walking: The multivariate adjusted odds ratio of developing MS after 10 years in participants free of MS at baseline was 0.51 (0.33–0.80) comparing a fast/very fast speed of walking with the reference of slow walking speed [32]. This could potentially explain a proportion of the association found between speed of walking and the risk of non-ischemic HF in the present study.

### Strengths and limitations

The strengths of this study include a large number of participants from the same geographical area, the prospective design, large number of outcomes, a sufficient follow-up time, detailed and updated registration of cardiovascular risk factors and complete follow-up regarding hospital admission and mortality. Results were only slightly attenuated by adjustment for confounders indicating that residual confounding is not likely to be of concern. Further, results were robust after a series of sensitivity analyses. The previous studies of PA and HF [12]–[18] have used only baseline data and despite prolonged follow-up have not taken changes in PA over time into account. This is likely to lead to misclassifications causing the true association to be underestimated; in the present study updated variables were used to reduce the probability of this misclassification.

Limitations of this study are that information on PA and duration of walking are self-reported, though identical or similar questionnaires been used in different studies [11], [16], [18], [33]. The intensity is reported in the form of relative intensity, which is considered a more appropriate measure than absolute intensity when the age-span is large and when the participants have wide differences in levels of physical fitness [11], [34]. The hospital discharge diagnosis of HF has been shown to have high specificity but low sensitivity [35] and we did not identify milder cases treated outside hospital. This will lead to an underestimation of HF but is not likely to bias the results

### Conclusion

This study shows an inverse dose-response relation between physical activity and subsequent HF and indicates that the intensity rather than duration of walking may be important. Current guidelines on primary and secondary prevention of CHD recommend 30 minutes per day of moderate or 20 minutes of strenuous exercise. The results suggest that the emphasis also in prevention of HF should be on intensity rather than duration of the exercise. One simple mean of reaching the goal is brisk walking and the higher the pace the greater the effect. The finding that the association between levels of physical activity and HF was not diminished after excluding a number of cases related to acute coronary syndrome suggest that the underlying mechanisms are not only through the well-established effects on risk of CHD but also have effect on non-ischaemic HF.

## Supporting Information

Table S1 **Hazard ratios for HF - Baseline data.** Analyses repeated using only baseline data (i.e. no time-dependent variables).
(DOCX)

Table S2 **Hazard ratios for HF – Reverse causation.** To avoid any influence of subclinical disease (reverse causation) the first two years of follow-up were excluded.
(DOCX)

Table S3 **Hazard ratios for HF – Competing risk.** Analysis allowing for the competing risk from mortality from any cause based on the method of Fine and Gray.
(DOCX)

Table S4 **Hazard ratios for HF – Death of HF before hospital admission censored.** Approximately 11% of cases of the outcomes were death from HF not preceded by hospital admission. Because the cause of death in this case is likely to be less valid, analyses were repeated treating these cases as censored.
(DOCX)

Table S5 **Hazard ratios for HF – Exclusion of participants reporting antihypertensive medication.**
(DOCX)

Table S6 **Hazard ratios for HF – Adjusted for proBNP.** Information on proBNP was available only from the fourth examination and analysis adjusting for proBNP was therefore repeated based only on this study.
(DOCX)
