# Supplementary material for: Speed and Duration of Walking and Other Leisure Time Physical Activity and the Risk of Heart Failure: A Prospective Cohort Study from the Copenhagen City Heart Study
Source: PLoS One. 2014 Mar 12;9(3):e89909. doi: 10.1371/journal.pone.0089909 (PMC3951187; doi:10.1371/journal.pone.0089909)
Supplement: Table S1 — Hazard ratios for HF - Baseline data. Analyses repeated using only baseline data (i.e. no time-dependent variables). (DOCX) [file pone.0089909.s001.docx]

**Analyses – baseline**

L**eisure-time physical activity – obus1-4.**

|  | **Age adjusted HR** | **HR^a^** | **HR^b^** |
| --- | --- | --- | --- |
| **Sedentary** | 1 (ref.) | 1 (ref.) | 1 (ref.) |
| **Light** | 0.67 (0.59-0.76) | 0.80 (0.70-0.91) | 0.85 (0.74-0.98) |
| **Moderate/High** | 0.66 (0.57-0.76) | 0.83 (0.72-0.96) | 0.91 (0.77 – 1.07) |
| *p-value* | *<0.001* | *0.03* | *0.29* |

^a^Adjusted for age and confounder included co-morbidity parameters as described in methods

^b^Adjusted for age, confounders (included co-morbidity parameters) and potential mediators as described in methods

**Intensity of walking – obus3-4.**

|  | **Age adjusted HR** | **HR^a^** | **HR^b^** |
| --- | --- | --- | --- |
| **Low** | 1 (ref.) | 1 (ref.) | 1 (ref.) |
| **Moderate** | 0.35 (0.29-0.43) | 0.47 (0.38-0.58) | 0.52 (0.42-0.65) |
| **High** | 0.18 (0.13-0.25) | 0.29 (0.21-0.41) | 0.37 (0.26-0.52) |
| *p-value* | *<0.001* | *<0.001* | *<0.001* |

^a^Adjusted for age and confounder included co-morbidity parameters as described in methods

^b^Adjusted for age, confounders (included co-morbidity parameters) and potential mediators as described in methods

**Duration of walking – obus3-4.**

|  | **Age adjusted HR** | **HR^a^** | **HR^b^** |
| --- | --- | --- | --- |
| **Never - ½ hour** | 1 (ref.) | 1 (ref.) | 1 (ref.) |
| **½ - 1 hour** | 0.70 (0.54-0.91) | 0.74 (0.56-0.96) | 0.76 (0.58-0.99) |
| **1 – 2 hours** | 0.74 (0.37-0.96) | 0.81 (0.67-1.06) | 0.85 (0.65-1.12) |
| **> 2 hours** | 0.73 (0.56-0.96) | 0.80 (0.61-1.04) | 0.85 (0.65-1.12) |
| *p-value* | *0.12* | *0.38* | *0.73* |

^a^Adjusted for age and confounder included co-morbidity parameters as described in methods

^b^Adjusted for age, confounders (included co-morbidity parameters) and potential mediators as described in methods
