# Supplementary material for: Speed and Duration of Walking and Other Leisure Time Physical Activity and the Risk of Heart Failure: A Prospective Cohort Study from the Copenhagen City Heart Study
Source: PLoS One. 2014 Mar 12;9(3):e89909. doi: 10.1371/journal.pone.0089909 (PMC3951187; doi:10.1371/journal.pone.0089909)
Supplement: Table S2 — Hazard ratios for HF – Reverse causation. To avoid any influence of subclinical disease (reverse causation) the first two years of follow-up were excluded. (DOCX) [file pone.0089909.s002.docx]

**Analyses – reverse causation**

L**eisure-time physical activity – obus1-4.**

|  | **Age adjusted HR** | **HR^a^** | **HR^b^** |
| --- | --- | --- | --- |
| **Sedentary** | 1 (ref.) | 1 (ref.) | 1 (ref.) |
| **Light** | 0.61 (0.51-0.74) | 0.76 (0.62-0.91) | 0.77 (0.64-0.93) |
| **Moderate/High** | 0.64 ( | 0.86 (0.71-1.04) | 0.89 (0.73-1.09) |
| *p-value* | *<0.001* | *0.37* | *0.62* |

^a^Adjusted for age and confounder included co-morbidity parameters as described in methods

^b^Adjusted for age, confounders (included co-morbidity parameters) and potential mediators as described in methods

**Intensity of walking – obus3-4.**

|  | **Age adjusted HR** | **HR** | **HR** |
| --- | --- | --- | --- |
| **Low** | 1 (ref.) | 1 (ref.) | 1 (ref.) |
| **Moderate** | 0.38 (0.28-0.52 | 0.48 (0.35-0.66) | 0.52 (0.37-0.71) |
| **High** | 0.17 (0.10-0.28) | 0.25 (0.15-0.44) | 0.30 (0.17-0.53) |
| *p-value* | *<0.001* | *<0.001* | *<0.001* |

^a^Adjusted for age and confounder included co-morbidity parameters as described in methods

^b^Adjusted for age, confounders (included co-morbidity parameters) and potential mediators as described in methods

**Duration of walking – obus3-4.**

|  | **Age adjusted HR** | **HR^a^** | **HR^b^** |
| --- | --- | --- | --- |
| **Never - ½ hour** | 1 (ref.) | 1 (ref.) | 1 (ref.) |
| **½ - 1 hour** | 0.80 (0.56-1.16) | 0.90 (0.62-1.31) | 0.90 (0.62-1.31) |
| **1 – 2 hours** | 0.69 (0.47-1.01) | 0.78 (0.52-1.15) | 0.78 (0.53-1.15) |
| **> 2 hours** | 1.06 (0.47-1.01) | 1.17 (0.81-1.70) | 1.17 (0.81-1.70) |
| *p-value* | *0.44* | *0.40* | *0.40* |

^a^ Adjusted for age and confounder included co-morbidity parameters as described in methods

^b^ Adjusted for age, confounders (included co-morbidity parameters) and potential mediators as described in methods
