# Supplementary material for: Speed and Duration of Walking and Other Leisure Time Physical Activity and the Risk of Heart Failure: A Prospective Cohort Study from the Copenhagen City Heart Study
Source: PLoS One. 2014 Mar 12;9(3):e89909. doi: 10.1371/journal.pone.0089909 (PMC3951187; doi:10.1371/journal.pone.0089909)
Supplement: Table S3 — Hazard ratios for HF – Competing risk. Analysis allowing for the competing risk from mortality from any cause based on the method of Fine and Gray. (DOCX) [file pone.0089909.s003.docx]

**Analyses – competing risk**

L**eisure-time physical activity – obus1-4.**

|  | **Age adjusted HR** | **HR^a^** | **HR^b^** |
| --- | --- | --- | --- |
| **Sedentary** | 1 (ref.) | 1 (ref.) | 1 (ref.) |
| **Light** | 0.69 (0.60-0.78) | 0.84 (0.73-0.96) | 0.88 (0.76-1.02) |
| **Moderate/High** | 0.70 (0.61-0.81) | 0.93 (0.80-1.07) | 1.00 (0.85.1.17) |
| *p-value* | *P<0.001* | *P=0.49* | *P=0.78* |

^a^Adjusted for age and confounder included co-morbidity parameters as described in methods

^b^Adjusted for age, confounders (included co-morbidity parameters) and potential mediators as described in methods

**Intensity of walking – obus3-4.**

|  | **Age adjusted HR** | **HR^a^** | **HR^b^** |
| --- | --- | --- | --- |
| **Low** | 1 (ref.) | 1 (ref.) | 1 (ref.) |
| **Moderate** | 0.46 (0.37-0.56) | 0.58 (0.46-0.72) | 0.63 (0.50-0.79) |
| **High** | 0.21 (0.15-0.30) | 0.32 (0.22-0.46) | 0.39 (0.26-0.57) |
| *p-value* | *P<0.001* | *P<0.001* | *P<0.001* |

^a^Adjusted for age and confounder included co-morbidity parameters as described in methods

^b^Adjusted for age, confounders (included co-morbidity parameters) and potential mediators as described in methods

**Duration of walking – obus3-4.**

|  | **Age adjusted HR** | **HR^a^** | **HR^b^** |
| --- | --- | --- | --- |
| **Never - ½ hour** | 1 (ref.) | 1 (ref.) | 1 (ref.) |
| **½ - 1 hour** | 0.75 (0.58-0.97) | 0.81 (0.62-1.07) | 0.84 (0.64-1.11) |
| **1 – 2 hours** | 0.73 (0.56-0.95) | 0.85 (0.64-1.11) | 0.90 (0.68-1.18) |
| **> 2 hours** | 0.86 (0.65-1.12) | 0.96 (0.73-1.27) | 1.02 (0.77-1.36) |
| *p-value* | *P=0.50* | *P=0.84* | *P=0.55* |

^a^Adjusted for age and confounder included co-morbidity parameters as described in methods

^b^Adjusted for age, confounders (included co-morbidity parameters) and potential mediators as described in methods
