# Supplementary material for: Speed and Duration of Walking and Other Leisure Time Physical Activity and the Risk of Heart Failure: A Prospective Cohort Study from the Copenhagen City Heart Study
Source: PLoS One. 2014 Mar 12;9(3):e89909. doi: 10.1371/journal.pone.0089909 (PMC3951187; doi:10.1371/journal.pone.0089909)
Supplement: Table S4 — Hazard ratios for HF – Death of HF before hospital admission censored. Approximately 11% of cases of the outcomes were death from HF not preceded by hospital admission. Because the cause of death in this case is likely to be less valid, analyses were repeated treating these cases as censored. (DOCX) [file pone.0089909.s004.docx]

**Analyses – excl. death of HF before hospital admission**

L**eisure-time physical activity – obus1-4.**

|  | **Age adjusted HR** | **HR^a^** | **HR^b^** |
| --- | --- | --- | --- |
| **Sedentary** | 1 (ref.) | 1 (ref.) | 1 (ref.) |
| **Light** | 0.61 (0.53-0.70) | 0.77 (0.67-0.88) | 0.82 (0.71-0.95) |
| **Moderate/High** | 0.60 (0.52-0.70) | 0.84 (0.72-0.98) | 0.93 (0.79-1.09) |
| *p-value* | *<0.001* | *0.07* | *0.61* |

^a^Adjusted for age and confounder included co-morbidity parameters as described in methods

^b^Adjusted for age, confounders (included co-morbidity parameters) and potential mediators as described in methods

**Intensity of walking – obus3-4.**

|  | **Age adjusted HR** | **HR^a^** | **HR^b^** |
| --- | --- | --- | --- |
| **Low** | 1 (ref.) | 1 (ref.) | 1 (ref.) |
| **Moderate** | 0.42 (0.34-0.52) | 0.47 (0.37-0.59) | 0.60 (0.47-0.75) |
| **High** | 0.19 (0.13-0.28) | 0.30 (0.21-0.43) | 0.39 (0.26-0.58) |
| *p-value* | *<0.001* | *<0.001* | *<0.001* |

^a^Adjusted for age and confounder included co-morbidity parameters as described in methods

^b^Adjusted for age, confounders (included co-morbidity parameters) and potential mediators as described in methods

**Duration of walking – obus3-4.**

|  | **Age adjusted HR** | **HR^a^** | **HR^b^** |
| --- | --- | --- | --- |
| **Never - ½ hour** | 1 (ref.) | 1 (ref.) | 1 (ref.) |
| **½ - 1 hour** | 0.79 (0.60-1.04) | 0.81 (0.61-1.08) | 0.82 (0.62-1.09) |
| **1 – 2 hours** | 0.79 (0.60-1.04) | 0.86 (0.66-1.15) | 0.90 (0.67-1.19) |
| **> 2 hours** | 0.76 (0.57-1.02) | 0.81 (0.60-1.08) | 0.85 (0.64-1.15) |
| *p-value* | *0.13* | *0.32* | *0.58* |

^a^Adjusted for age and confounder included co-morbidity parameters as described in methods

^b^Adjusted for age, confounders (included co-morbidity parameters) and potential mediators as described in methods
