# Supplementary material for: Speed and Duration of Walking and Other Leisure Time Physical Activity and the Risk of Heart Failure: A Prospective Cohort Study from the Copenhagen City Heart Study
Source: PLoS One. 2014 Mar 12;9(3):e89909. doi: 10.1371/journal.pone.0089909 (PMC3951187; doi:10.1371/journal.pone.0089909)
Supplement: Table S5 — Hazard ratios for HF – Exclusion of participants reporting antihypertensive medication. (DOCX) [file pone.0089909.s005.docx]

**Analyses – after exclusion of participants reporting antihypertensive treatment**

**Intensity of walking – obus3-4.**

|  | **Age adjusted HR** | **HR^a^** | **HR^b^** |
| --- | --- | --- | --- |
| **Low** | 1 (ref.) | 1 (ref.) | 1 (ref.) |
| **Moderate** | 0.41 (0.32-0.52) | 0.52 (0.40-0.67) | 0.59 (0.45-0.77) |
| **High** | 0.19 (0.13-0.29) | 0.30 (0.19-0.45) | 0.38 (0.25-0.59) |
| *p-value* | *<0.001* | *<0.001* | *<0.001* |

^a^Adjusted for age and confounder included co-morbidity parameters as described in methods

^b^Adjusted for age, confounders (included co-morbidity parameters) and potential mediators as described in methods

**Duration of walking – obus3-4.**

|  | **Age adjusted HR** | **HR^a^** | **HR^b^** |
| --- | --- | --- | --- |
| **Never - ½ hour** | 1 (ref.) | 1 (ref.) | 1 (ref.) |
| **½ - 1 hour** | 0.70 (0.52-0.96) | 0.78 (0.56-1.07) | 0.82 (0.59-1.13) |
| **1 – 2 hours** | 0.72 (0.53-0.98) | 0.86 (0.63-1.19) | 0.95 (0.68-1.31) |
| **> 2 hours** | 0.95 (0.70-1.29) | 1.07 (0.78-1.48) | 1.19 (0.86-1.64) |
| *p-value* | *0.70* | *0.24* | *0.08* |

^a^Adjusted for age and confounder included co-morbidity parameters as described in methods

^b^Adjusted for age, confounders (included co-morbidity parameters) and potential mediators as described in methods
