# Supplementary material for: Speed and Duration of Walking and Other Leisure Time Physical Activity and the Risk of Heart Failure: A Prospective Cohort Study from the Copenhagen City Heart Study
Source: PLoS One. 2014 Mar 12;9(3):e89909. doi: 10.1371/journal.pone.0089909 (PMC3951187; doi:10.1371/journal.pone.0089909)
Supplement: Table S6 — Hazard ratios for HF – Adjusted for proBNP. Information on proBNP was available only from the fourth examination and analysis adjusting for proBNP was therefore repeated based only on this study. (DOCX) [file pone.0089909.s006.docx]

**Analyses – examination 4 – additional adjustment for proBNP**

**Intensity of walking – obus4.**

|  | **Age adjusted HR** | **HR^a^** | **HR^b^** |
| --- | --- | --- | --- |
| **Low** | 1 (ref.) | 1 (ref.) | 1 (ref.) |
| **Moderate** | 0.31 (0.21-0.48) | 0.41 (0.27-0.63) | 0.53 (0.34-0.84) |
| **High** | 0.11 (0.05-0.24) | 0.18 (0.08-0.41) | 0.24 (0.100.58) |
| *p-value* | *<0.001* | *<0.001* | *<0.001* |

^a^Adjusted for age and confounder included co-morbidity parameters as described in methods

^b^Adjusted for age, confounders (included co-morbidity parameters) and potential mediators as described in methods (+proBNP)

**Duration of walking – obus4.**

|  | **Age adjusted HR** | **HR^a^** | **HR^b^** |
| --- | --- | --- | --- |
| **Never - ½ hour** | 1 (ref.) | 1 (ref.) | 1 (ref.) |
| **½ - 1 hour** | 0.87 (0.53-0.42) | 1.05 (0.63-1.77) | 1.24 (0.72-2.15) |
| **1 – 2 hours** | 0.64 (0.38-1.08) | 0.75 (0.43-1.30) | 0.91 (0.51-1.63) |
| **> 2 hours** | 0.70 (0.38-1.29) | 0.66 (0.34-1.29) | 0.76 (0.38-1.52) |
| *p-value* | *0.10* | *0.08* | *0.22* |

^a^Adjusted for age and confounder included co-morbidity parameters as described in methods

^b^Adjusted for age, confounders (included co-morbidity parameters) and potential mediators as described in methods (+proBNP)
